# Inter-Organellar Ca^2+^ Homeostasis in Plant and Animal Systems

**DOI:** 10.3390/cells14151204

**Published:** 2025-08-06

**Authors:** Philip Steiner, Susanna Zierler

**Affiliations:** 1 Institute of Pharmacology, Faculty of Medicine, Johannes Kepler University Linz, 4020 Linz, Austria; 2 Walther Straub Institute of Pharmacology and Toxicology, Ludwig-Maximilians-Universität München, 80336 Munich, Germany

**Keywords:** calcium homeostasis, organelle communication, IP3 receptors, ryanodine receptors, two-pore channels, mitochondrial calcium uniporters, TRPML channels, membrane contact sites, animal calcium signaling, plant calcium signaling

## Abstract

The regulation of calcium (Ca^2+^) homeostasis is a critical process in both plant and animal systems, involving complex interplay between various organelles and a diverse network of channels, pumps, and transporters. This review provides a concise overview of inter-organellar Ca^2+^ homeostasis, highlighting key regulators and mechanisms in plant and animal cells. We discuss the roles of key Ca^2+^ channels and transporters, including IP_3_Rs, RyRs, TPCs, MCUs, TRPMLs, and P2XRs in animals, as well as their plant counterparts. Here, we explore recent innovations in structural biology and advanced microscopic techniques that have enhanced our understanding of these proteins’ structure, functions, and regulations. We examine the importance of membrane contact sites in facilitating Ca^2+^ transfer between organelles and the specific expression patterns of Ca^2+^ channels and transporters. Furthermore, we address the physiological implications of inter-organellar Ca^2+^ homeostasis and its relevance in various pathological conditions. For extended comparability, a brief excursus into bacterial intracellular Ca^2+^ homeostasis is also made. This meta-analysis aims to bridge the gap between plant and animal Ca^2+^ signaling research, identifying common themes and unique adaptations in these diverse biological systems.

## 1. Introduction

Calcium (Ca^2+^) is a ubiquitous second messenger that plays an important role in numerous cellular processes in both plant and animal systems. What makes Ca^2+^ such a powerful second messenger is the enormous chemical gradient between the extracellular free Ca^2+^ concentration, ranging between 0.8 and 2 mM, and cytosolic free Ca^2+^ concentrations of resting cells, which can be as low as 100 nM, which is ten-thousand times lower [1,2,3,4,5]. The maintenance of Ca^2+^ homeostasis is essential for proper cellular function and involves manifold interplay between various organelles [6]. In eukaryotic cells, the endoplasmic reticulum (ER) serves as the primary intracellular Ca^2+^ store, while mitochondria, chloroplasts (in plants), and other organelles also contribute to Ca^2+^ buffering and signaling. The coordinated efforts of these organelles in regulating Ca^2+^ levels are critical for cell survival, growth, and response to environmental stimuli. Calmodulin (CaM) serves as a ubiquitously conserved Ca^2+^ sensor in both animal and plant cells, but shows a pronounced functional diversification [7,8]. While animal CaM has a uniform sequence, plants express different CaM isoforms. CaM-like proteins (CMLs) are a plant-specific expansion of Ca^2+^ sensors, which are not present in animal cells [9,10,11]. CMLs comprise unique expression patterns and target specificities, providing plants with specialized Ca^2+^ signaling capabilities for environmental adaptation [12,13]. Troponin C (TnC) is predominantly restricted to animal muscle cells, where it regulates contraction through Ca^2+^-dependent conformational changes, with no functional equivalent in plant cells [14]. Ca^2+^-dependent protein kinases (CDPKs) are ubiquitously expressed in plant cells and some protozoans but not in animal cells [15,16]. CDPKs combine Ca^2+^ sensing and kinase activity within a single polypeptide, enabling direct phosphorylation responses to Ca^2+^ signals without requiring separate CaM intermediates [17,18]. Recent advances in imaging techniques, ultra-resolution microscopy and molecular tools have significantly enhanced our understanding of the complex mechanisms underlying interorganellar Ca^2+^ homeostasis in both plant and animal systems [19,20,21,22,23]. The diverse network of Ca^2+^ signaling pathways involves numerous proteins, including channels, pumps, and exchangers, which work in concert to maintain precise spatiotemporal control over intracellular Ca^2+^ concentrations. In animal cells, the sarco/endoplasmic reticulum Ca^2+^-ATPase (SERCA) actively pumps Ca^2+^ into the ER lumen, while inositol 1,4,5-trisphosphate receptors (IP_3_Rs) and ryanodine receptors (RyRs) mediate Ca^2+^ release from the ER [24,25]. Similarly, in plant cells, Ca^2+^-ATPases and intracellular ion channels such as two-pore channels (TPCs) play crucial roles in maintaining Ca^2+^ gradients across organelle membranes [26,27,28]. In this review, we did not focus on voltage-gated Ca^2+^ channels as they were extensively reviewed before [29,30,31]. Mitochondria, with their ability to rapidly uptake and release Ca^2+^, act as important modulators of cytosolic Ca^2+^ signals and contribute to the shaping of Ca^2+^ microdomains [32,33]. Interorganellar communication, not only between the ER and mitochondria but also between the ER and the endo-lysosomal compartment, has emerged as a critical aspect of Ca^2+^ homeostasis. Membrane contact sites (MCS) between these organelles facilitate the efficient transfer of Ca^2+^ and other molecules, allowing for fine-tuned regulation of cellular processes [24,34,35,36,37,38,39]. In recent years, the discovery of proteins such as PDZD8, which were shown to tether ER and mitochondria in mammalian neurons, has shed light on the molecular mechanisms underlying these interactions [40]. Moreover, the store-operated Ca^2+^ entry (SOCE) pathway, mediated by STIM and ORAI proteins, plays a vital role in replenishing ER Ca^2+^ stores and maintaining long-term Ca^2+^ homeostasis and signaling in various cell types, including immune cells [41,42]. In plant systems, the presence of chloroplasts adds another layer of complexity to Ca^2+^ signaling networks. These organelles not only participate in photosynthesis but also contribute to Ca^2+^ homeostasis and signaling, particularly in response to light and other environmental cues [43]. The evolution of Ca^2+^-based signaling in plants has led to the development of unique mechanisms for integrating information from various cellular compartments and responding to diverse stimuli [44,45]. This review aims to provide a comprehensive overview of the current knowledge regarding the key regulators involved in maintaining Ca^2+^ balance across different cellular compartments, highlighting both similarities and differences between plant and animal systems. We will explore the latest findings on interorganellar Ca^2+^ homeostasis, discuss the physiological implications of these mechanisms, and identify areas for future research in this rapidly evolving field.

## 2. Key Players in Intracellular Ca^2+^ Regulation in Animal Cells

**Figure 1:**
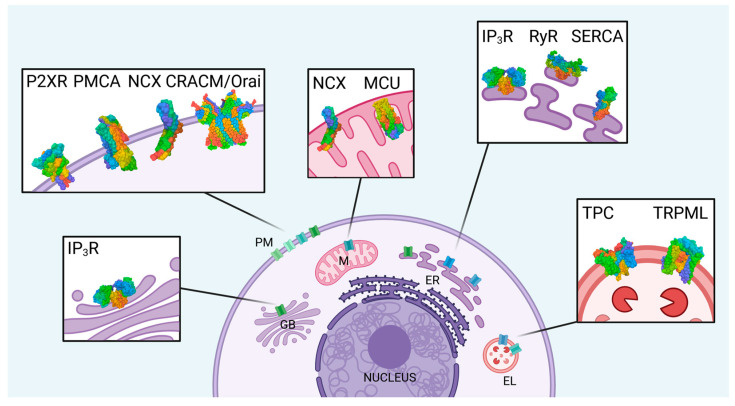
Key regulators for intracellular Ca^2+^ homeostasis in animal cells. The illustration indicates key components of intracellular Ca^2+^ regulation across various cellular compartments. The endoplasmic reticulum (ER), mitochondria (M), and the endolysosomal (EL) compartment serve as Ca^2+^ stores. Ca^2+^ release from the ER is mediated by inositol 1,4,5-trisphosphate receptors (IP_3_R) and ryanodine receptors (RyR), while Ca^2+^ uptake is facilitated by sarco/endoplasmic reticulum Ca^2+^-ATPase (SERCA). The mitochondrial calcium uniporter (MCU) enables rapid Ca^2+^ uptake into mitochondria. Two-pore channels (TPC) and transient receptor potential mucolipin (TRPML) channels regulate Ca^2+^ flux in EL. P2X receptors (P2XR) act as ligand-gated ion channels in the plasma membrane (PM). PM Ca^2+^ ATPase (PMCA), Ca^2+^ release-activated Ca^2+^ channels (CRACM or Orai), and Na^+^/Ca^2+^ exchanger (NCX) maintain cytosolic Ca^2+^ levels. IP_3_Rs are also present in Golgi bodies (GB), contributing to local Ca^2+^ signaling. Created with biorender.com. PDB data were also generated using the PDB tool from biorender.com and show a schematic of the van der Waals structure with color codes corresponding to the different sequences. Localization and orientation correspond to the known nature of the proteins.

The following chapter summarizes the most important intracellular ion channels and transporters regulating Ca^2+^ homeostasis and signaling in animal cells. The intracellular localization of the individual channels and transporters is schematically depicted in Figure 1, showing the known predominant position of the channels in the various compartment membranes, including the known protein data bank (PDB) structures.

### 2.1. Inositol Trisphosphate Receptors

Inositol 1,4,5-trisphosphate receptors (IP_3_Rs) are crucial intracellular Ca^2+^ channels that play a vital role in cellular signaling and Ca^2+^ homeostasis in animal cells. Recent structural studies have provided new insights into the activation mechanisms of IP_3_Rs [46]. These receptors are primarily localized in the ER but can also reside in the biomembrane of Golgi bodies (Figure 1; PDB: 8TL9) and regulate Ca^2+^ release in response to IP_3_ and local Ca^2+^ increase due to interplay with other ion channels [37,38,46,47,48]. IP_3_Rs are involved in various physiological processes, including cell proliferation, smooth muscle contraction, apoptosis, and gene expression [49,50,51]. Moreover, dysregulation of IP_3_Rs has been implicated in several pathological conditions, such as neurodegenerative diseases and cancer [24,51]. The spatial organization of IP_3_Rs within cells has been shown to be critical for the propagation of Ca^2+^ signals and the selective regulation of cellular responses [47,52].

### 2.2. Ryanodine Receptors

Ryanodine receptors (RyRs) are essential intracellular Ca^2+^ release channels located in the sarcoplasmic/endoplasmic reticulum (Figure 1; PDB: 8UQ4) of various animal cells. RyRs are regulated by a complex interplay of Ca^2+^ concentrations, redox state, small molecules, and protein interactions and primarily conduct Ca^2+^ [53,54,55]. These homotetrameric proteins are important for numerous cellular processes, including excitation–contraction coupling in muscle cells and neurotransmission in neurons [56,57]. RyR1, for example, directly interacts with dihydropyridine receptors (DHPRs) through physical coupling mechanisms in skeletal muscle, where tetrads of DHPRs bind to RyR1s in an alternating manner and form two distinct connection sites between the channels without requiring Ca^2+^ influx [58]. In contrast, RyR2 is generally activated by Ca^2+^ itself through Ca^2+^-induced Ca^2+^ release (CICR) in cardiac muscle, where Ca^2+^ influx via L-type Ca^2+^ channels triggers RyR2 opening by binding to Ca^2+^-sensing sites on the receptor [59,60,61]. Previous investigations have revealed the complex structure–function relationship of RyRs, with super-resolution microscopy techniques identifying distinct subpopulations of RyR clusters in cardiac cells [21,22]. The spatial organization and interactions of these clusters significantly influence Ca^2+^ signaling dynamics and cellular function. Furthermore, research has highlighted the involvement of RyRs in pathological conditions, such as their potential role in Alzheimer’s disease [62].

### 2.3. Two-Pore Channels

Two-pore channels (TPCs) are intracellular voltage- and ligand-gated cation channels located in the endo-lysosomal system (Figure 1; PDB: 6NQ0) of animal cells [63]. In most vertebrates, all three TPC variants—TPC1, TPC2, and TPC3—are expressed, except in humans, mice, and rats, where TPC3 is not present [64,65,66,67]. These channels play crucial roles in various cellular processes, including Ca^2+^ signaling, membrane trafficking, and organelle morphology [39]. Structural and functional studies have revealed that TPCs are regulated by multiple factors, including the second messenger NAADP, indirectly via JPT2 and LSM12, phosphoinositides such as PI(3,5)P_2_, and voltage [68,69,70]. In this context, it was hypothesized that upon activation via NAADP, TPCs conduct primarily Ca^2+^ and upon activation via PI(3,5)P_2_, they conduct primarily Na^+^ [68,69,70]. Furthermore, researchers have uncovered novel functions of TPCs in regulating inter-organellar communication, particularly between the ER and other organelles such as the apicoplast in parasites [71] or endo-lysosomes in mice [38]. The interplay between TPC2 and IP_3_Rs to coordinate Ca^2+^ signals between lysosomes and the ER has also been recently described [38,48] but TPC1 has also been shown to regulate ER–endo-lysosome Ca^2+^ homeostasis in certain cell types [38]. TPCs have emerged as important players in several pathophysiological conditions, including Parkinson’s disease, non-alcoholic fatty liver disease, anaphylaxis, Ebola infection, cancer, cardiac dysfunction, and diabetes [38,72]. The complex regulation and diverse functions of TPCs make them promising targets for therapeutic interventions in various diseases.

### 2.4. Stromal Interaction Molecules, Orai Proteins, and Store-Operated Calcium Entry

In animal cells, Stromal Interaction Molecules (STIM) and Orai or CRACM proteins play crucial roles in regulating intracellular Ca^2+^ levels and signaling (Figure 1; PDB: 7KR5). STIM proteins (STIM1, STIM2), located in the ER, sense decreases in luminal Ca^2+^ and activate Orai channels (Orai1, Orai2, Orai3) in the plasma membrane to facilitate Ca^2+^ influx [73,74,75,76,77]. This process, known as store-operated calcium entry (SOCE), is essential for various cellular functions, including cell growth and immune responses [78]. The interaction between STIM and Orai is highly specific, with STIM binding to Orai via conserved domains to activate CRAC channels [79,80]. This interaction is critical for maintaining Ca^2+^ homeostasis and modulating downstream signaling pathways [81]. Dysregulation of CRAC channels has been linked to immune deficiencies and other diseases, highlighting their importance in cellular function [73,74,82,83,84].

### 2.5. Mitochondrial Calcium Uniporters

The mitochondrial calcium uniporter (MCU), located in the inner mitochondrial membrane (Figure 1; PDB: 6XJV), is a highly selective Ca^2+^ channel complex that plays a crucial role in regulating intracellular Ca^2+^ signaling, bioenergetics and cell death in animal cells [85]. Previously, it was shown that the MCU complex consists of multiple subunits, including the pore-forming MCU protein, its paralog MCUb, the essential MCU regulator (EMRE), and regulatory MICU proteins in the intermembrane space [86,87]. The complex interplay between these components allows for precise control of mitochondrial Ca^2+^ uptake, with MICU1 and MICU2 acting as gatekeepers and regulators by sensing cytosolic Ca^2+^ to prevent Ca^2+^ overload under resting conditions while facilitating rapid uptake during signaling events [32,88,89]. Interestingly, the activity and expression of MCU varies significantly among different tissues, with cardiac mitochondria exhibiting surprisingly low MCU current density, likely to prevent excessive Ca^2+^ uptake in this highly metabolically active tissue [90]. In addition to that, previous studies have highlighted the importance of MCU in various physiological and pathological processes, including T-cell-mediated inflammation, cancer cell migration, and cardiac function, making MCU an essential modulator for future biomedical research [91,92,93].

### 2.6. Transient Receptor Potential Channels

Transient receptor potential (TRP) channels are a diverse superfamily of cation permeable ion channels, classified into six subfamilies (TRPC, TRPV, TRPM, TRPA, TRPP and TRPML) [94]. Two of these subfamilies, are (also) expressed on organelles and thus described in more detail below: TRPML and TRPM. The transient receptor potential mucolipin (TRPML) subfamily comprises three members (TRPML1, TRPML2, and TRPML3) in mammals, encoded by MCOLN1-3 genes. These non-selective cation channels are primarily localized in the endolysosomal system of animal cells (Figure 1; PDB: 6E7Z), playing pivotal roles in various cellular processes [95]. TRPML channels are regulated by multiple factors, including phosphoinositides, reactive oxygen species (ROS), and pH, with PI(3,5)P_2_ acting as an endogenous activator and PI(4,5)P_2_ as an inhibitor [96]. Previous investigations shed light on the complex regulatory mechanisms of TRPMLs, with cryo-electron microscopy providing insights into their molecular architecture [23]. TRPML1, the best-characterized member, functions as an “ROS sensor” and is involved in lysosomal Ca^2+^ release, autophagy regulation, and maintenance of cellular redox homeostasis [97]. TRPMLs have been implicated in several pathological conditions, including neurodegenerative disorders such as Alzheimer’s and Parkinson’s diseases, immune diseases as well as various forms of cancer [96,98,99,100,101,102,103,104]. Due to their involvement in numerous human diseases, TRPMLs are emerging as potential targets for the development of new therapeutic strategies and treatments [105]. Transient receptor potential melastatin-like (TRPM) channels are classified into TRPM1-7. TRPM2, for example, plays an important role in lysosomal Ca^2+^ release in dendritic cells and beta cells [106,107]. Several studies have examined the role of the unique channel-kinase TRPM7 within various vesicular compartments [108,109,110,111], highlighting its possible participation in vesicular Ca^2+^ signaling. As it also harbors a functional serine/threonine kinase, it may contribute to cellular signaling via modulating other signaling proteins [112,113,114]. Additional research is required to clarify exact molecular mechanisms.

### 2.7. P2X Receptors

P2X receptors (P2X1-7) are trimeric ATP-gated cation channels that play crucial roles in diverse cellular processes across animal cell types and are mainly located in the plasma membrane (Figure 1; PDB: 8JV8). Recent cryo-electron microscopy studies have depicted detailed structures of P2X receptors, elucidating mechanisms of agonist binding and channel gating [115,116,117]. P2X receptors are Ca^2+^-permeable and can be modulated by extracellular Na^+^, Mg^2+^, Ca^2+^, and H^+^. P2X receptors, particularly P2X7, are implicated in numerous physiological and pathophysiological conditions, including neurodegenerative diseases, inflammation, and pain [118,119,120,121]. The expression and function of P2X receptors vary across cell types, with P2X7 predominantly expressed in immune and glial cells [122,123,124,125]. Recently, the potential of P2X receptors as therapeutic targets was discussed, with the development of subtype-specific modulators showing promise in preclinical models [126,127]. In the study of Poejo et al., it was shown that Preyssler-type polyoxotungstate (P5W30) acts as a novel agonist of purinergic P2 receptors, inducing dose-dependent increases in cytosolic Ca^2+^ concentration in mouse hippocampal neuronal cells primarily through metabotropic receptor activation, suggesting its potential as a therapeutic tool for targeting purinergic signaling pathways [128]. In addition to that, the discovery of splice variants, especially for P2X7, has added additional complexity to P2X receptor biology and pharmacology [122,129].

### 2.8. Sarco/Endoplasmic Reticulum Ca^2+^-ATPase

The sarco/endoplasmic reticulum Ca^2+^-ATPase (SERCA; SERCA1-3 isoforms), located in the ER membrane (Figure 1; PDB: 3B9R), is an important regulator for intracellular Ca^2+^ homeostasis in animal cells [130]. Studies have highlighted SERCA’s importance in various physiological processes and pathological conditions. In cardiac tissue, SERCA2a gene therapy has shown promise in treating heart failure, improving contractility and survival in animal models [131]. SERCA dysfunction has also been implicated in neurodegenerative diseases [132]. The pump’s activity is regulated by phospholamban and sarcolipin in muscle cells [133]. SERCA can be targeted in cancer cells by small molecules and pharmacological compounds such as thapsigargin (and prodrug derivatives) which disrupt Ca^2+^ balance and trigger programmed cell death [134,135,136,137]. This makes SERCA an important target for cancer research. It was also shown, that polyoxotungstates (POTs), particularly Wells–Dawson POTs and Preyssler-type anions (see above), are potential inhibitors of SERCA [138]. Recent structural studies have provided insights into SERCA’s molecular mechanisms, such as investigating the molecular architecture underlying the diverse roles of structurally similar SERCA modulators [139], while its role in cellular processes like autophagy and ER stress response has been largely elucidated [140].

### 2.9. Plasma Membrane Calcium ATPase

The plasma membrane Ca^2+^-ATPase (PMCA; Figure 1; PDB: 6A69) is essential for regulating and maintaining the balance of Ca^2+^ ions within animal cells, playing a vital role in intracellular Ca^2+^ homeostasis [141]. It was shown that PMCA is involved in various physiological processes, including neuronal signaling, cardiac function, and cancer progression [142]. PMCA isoforms (PMCA1-4) exhibit tissue-specific expression patterns and are regulated by CaM and other signaling molecules [143]. Targeting PMCA function has emerged as a potential therapeutic approach for various diseases such as deafness, neurological disorders, autism, or cardiovascular diseases and cancer [142,144,145].

### 2.10. Sodium–Calcium Exchanger

The sodium–calcium exchanger (NCX; NCX1-3) is important for maintaining intracellular Ca^2+^ homeostasis in animal cells. As an antiporter membrane protein, NCX exports one Ca^2+^ ion in exchange for three Na^+^ ions, utilizing the electrochemical gradient of Na^+^ [146]. NCX is essential for various cellular functions, including neurosecretion, cardiac muscle relaxation, and maintaining low intracellular Ca^2+^ levels [147,148]. NCXs are primarily found in the plasma membrane [149,150], but isoforms can also be found in the mitochondrial membrane (Figure 1; PDB: 3US9) [151,152]. Also, NCXs’ involvement in Ca^2+^ signaling pathways and its potential as a therapeutic target in cardiovascular and neurological disorders was highlighted before [148,153].

## 3. Key Players in Intracellular Ca^2+^ Regulation in Plant Cells

**Figure 2:**
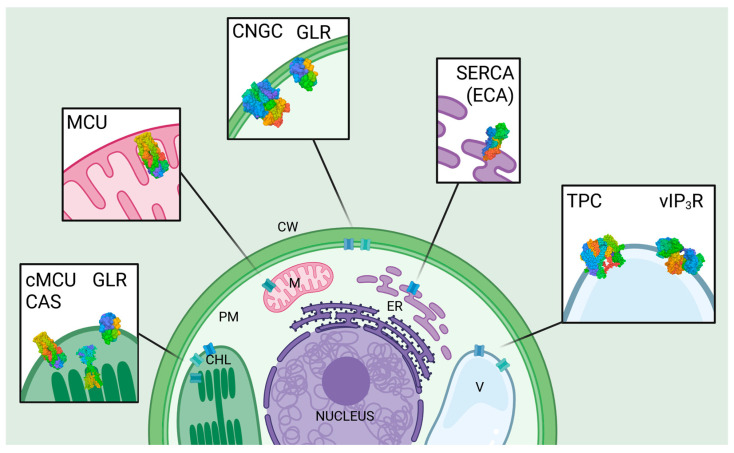
Key regulators for intracellular Ca^2+^ homeostasis in plant cells. Ca^2+^ homeostasis in plant cells involves the vacuole (V), ER, chloroplast (CHL), and cell wall (CW) as major Ca^2+^ stores. SERCA-like pumps (ECA) pump Ca^2+^ into the ER. Cyclic nucleotide-gated channels (CNGC) and glutamate receptor-like channels (GLR) facilitate Ca^2+^ influx across the plasma (and chloroplast) membrane. Two-pore channels (TPC) mediate Ca^2+^ release from the vacuole. Mitochondrial calcium uniporters (MCU) allow Ca^2+^ uptake into mitochondria, although their presence is less well-characterized than in animal cells. While not fully characterized in plants, recent studies suggest the potential existence of IP_3_ receptor-like channels in the vacuole (vIP_3_R), contributing to Ca^2+^ release. The chloroplast has an important yet special function for intracellular, intraorganellar, and plastidial Ca^2+^ homeostasis, with chloroplast MCU (cMCU), GLRs, and Ca^2+^-sensing receptors (CAS) highlighted in this scheme. Created with biorender.com; PDB data were also generated using the PDB tool from biorender.com and show a schematic of the van der Waals structure with color codes corresponding to the different sequences. Localization and orientation correspond to the known nature of the proteins.

This section provides an overview of the key players involved in intracellular Ca^2+^ regulation within plant cells, highlighting the most important Ca^2+^ channels and transporters. The intracellular localization of the individual channels and transporters is schematically depicted in Figure 2, showing the known predominant position of the channels in the various compartment membranes, including the known or related protein data bank (PDB) structures.

### 3.1. Vacuolar Inositol Trisphosphate Receptor

Vacuolar inositol trisphosphate receptors (vIP_3_R) might play a crucial role in intracellular Ca^2+^ homeostasis in plant cells. These receptors are thought to be Ca^2+^-permeable channels located in the vacuolar membrane (tonoplast; Figure 2; PDB: 8TL9, human structure schematically used for visualization due to the lack of plant PDB data), activated by IP_3_ as a second messenger [154,155,156]. However, their existence and properties in plants remain controversial, with limited homology to animal counterparts [155]. So far, no analogs of mammalian-like IP_3_Rs could be characterized in plant cells. Presumably located in the vacuolar membrane, vIP_3_Rs are thought to mediate Ca^2+^-induced Ca^2+^ release (CICR) mechanisms essential for cellular Ca^2+^ homeostasis [155,157,158]. They might play a pivotal role in intracellular Ca^2+^ regulation by facilitating controlled Ca^2+^ efflux from the massive vacuolar Ca^2+^ stores—which can contain Ca^2+^ concentrations up to three orders of magnitude higher than cytosolic levels and thereby enabling precise temporal and spatial control of cytosolic Ca^2+^ signatures required for diverse physiological processes including stress responses, developmental regulation, and signal transduction pathways [157,159,160,161]. Despite the potential importance of these receptors, their molecular identity and exact mechanisms in plants are still not fully understood, highlighting the need for further research in this area [155].

### 3.2. Cyclic Nucleotide-Gated Channels

Located in the plasma membrane of plant cells (Figure 2; PDB: 9J34), cyclic nucleotide-gated channels (CNGCs) play an important role in maintaining intracellular Ca^2+^ homeostasis [162]. These ion channels, regulated by cyclic nucleotides such as cAMP and cGMP, form a complex network with Ca^2+^ signaling pathways [163]. CNGCs are involved in various physiological processes, including defense responses, abiotic stress adaptation, and development [164]. Previous investigations have elucidated that CNGCs contribute to Ca^2+^ influx across the plasma membrane, thereby modulating cytosolic Ca^2+^ levels [165]. The interaction between CNGCs and Ca^2+^ is bidirectional, with Ca^2+^-CaM binding regulating channel activity [166]. Furthermore, the crosstalk between cyclic nucleotides and Ca^2+^ signaling has been observed in multiple plant species and cell types, highlighting its importance in plant physiology [163]. Understanding CNGC function and regulation is crucial for deciphering plant responses to environmental stimuli and developmental cues.

### 3.3. Two-Pore Channels

TPCs play a crucial role in intracellular Ca^2+^ homeostasis in plant cells. These voltage-dependent ion channels are located in the vacuolar membrane (Figure 2; PDB: 5DQQ) and mediate Ca^2+^ release from intracellular stores [27,28]. Compared to its animal counterpart, only TPC1 is relatively well described in plant cells [167,168]. TPCs are involved in various physiological processes, including stress responses and stomatal regulation [169]. Furthermore, TPCs contribute to the generation of specific Ca^2+^ signatures that encode information during plant–microbe interactions and abiotic stress responses [170]. Another important innovation is the high-resolution atomic representation of TPC1 in plant cells, which provides insights into the structure but also allows interpretation for potential functionality [171,172,173]. In Guo et al. [174], it was shown that Arabidopsis TPC (AtTPC1) favors Ca^2+^ and is nonselective among monovalent cations, whereas human TPC (HsTPC2) is PIP_2_-activated and thought to be Na^+^-selective. There it was shown that replacing three filter residues in AtTPC1 confers Na^+^ selectivity, revealing the structural determinants that drive divergent ion preference in TPCs despite their similar filter sequences. TPCs therefore undoubtedly play an important role in Ca^2+^ regulation in plant cells.

### 3.4. Mitochondrial Calcium Uniporters

MCUs are located in the inner mitochondrial membrane (Figure 2; PDB: 6DT0, fungus structure schematically used for visualization due to the lack of plant PDB data) and are critical intracellular Ca^2+^ homeostasis regulators in plant cells. Recent studies have demonstrated that plant MCU proteins, similar to their mammalian counterparts, mediate mitochondrial Ca^2+^ transport and represent the major route for rapid Ca^2+^ uptake [33,175]. The MCU complex in plants consists of pore-forming subunits and regulatory proteins, including MICU, which modulates the channel activity [176]. Research has shown that AtMCU1, an Arabidopsis MCU isoform, forms a Ca^2+^-permeable channel sensitive to known inhibitors and MICU regulation [175]. Impairment of MCU function in plants affects mitochondrial Ca^2+^ dynamics, ultrastructure, and root growth under certain conditions [175]. Furthermore, MCU-mediated mitochondrial Ca^2+^ transport has been linked to phytohormone signaling and thigmomorphogenesis, highlighting its importance in plant development and environmental responses [33].

### 3.5. SERCA-like Transporters/ER-Type Ca^2+^ ATPases

Plant cells contain specialized, SERCA-like Ca^2+^ transporters (Figure 2; PDB: 3B9R, rabbit structure schematically used for visualization due to the lack of plant PDB data) known as ER-type Ca^2+^ ATPases (ECAs) that play crucial roles in maintaining intracellular Ca^2+^ homeostasis [177]. These P-type IIA Ca^2+^ ATPases are evolutionarily related to animal SERCA and share approximately 50–53% amino acid sequence identity with their animal counterparts [177,178]. Plant ECAs are primarily localized to the ER membrane and function as ATP-driven Ca^2+^ pumps that transport Ca^2+^ from the cytosol into the ER lumen, thereby maintaining the low cytosolic Ca^2+^ concentrations (0.1–0.2 µM) essential for cellular function [179]. Unlike animal SERCA pumps, plant ECAs are typically insensitive to CaM regulation and show distinct pharmacological properties, being inhibited by cyclopiazonic acid but not by thapsigargin [177,180]. It was shown that plant ECAs, particularly ECA1 in Arabidopsis, are essential for stress tolerance, pollen fertility, and proper Ca^2+^ signaling responses to environmental stimuli, with their activity being regulated by Ca^2+^-dependent protein kinases (CPKs) that phosphorylate and activate these pumps during osmotic stress [181,182]. However, research on SERCA-like transporters in plant cells has made little progress in recent years compared to their animal counterparts [183].

### 3.6. Glutamate Receptor-like Channels

Plant glutamate receptor-like channels (GLRs) predominantly reside in the plasma membrane but can also be located in the chloroplast membrane (Figure 2; PDB: 6R85) and contribute to the regulation of intracellular Ca^2+^ homeostasis and signaling in plant cells [184]. These transmembrane proteins allow the movement of various ions across membranes, particularly Ca^2+^, which act as a key second messenger in plant responses to both endogenous and exogenous stimuli [184]. GLRs are involved in numerous physiological processes, including pollen development, sexual reproduction, root development, stomatal regulation, and pathogen response [184]. Recent studies have demonstrated their importance in long-distance electrical and Ca^2+^ signaling [185]. GLRs mediate Ca^2+^ influx in response to amino acids and other stimuli, generating specific Ca^2+^ signatures that are decoded by Ca^2+^-sensing proteins to initiate downstream signaling cascades [185,186]. Understanding GLR function is crucial for elucidating plant stress responses and developmental adaptations to changing environments [187].

### 3.7. Chloroplast

The chloroplast plays an important and exceptional role as an organelle in intracellular Ca^2+^ regulation and signaling in plant cells (Figure 2). It can generate specific stromal Ca^2+^ regulation in response to environmental stimuli [43]. Several Ca^2+^-permeable channels and transporters in chloroplast membranes have been identified, including the chloroplast-localized mitochondrial calcium uniporter (cMCU; Figure 2; PDB: 6DT0, fungus structure schematically used for visualization due to the lack of plant PDB data), which mediates Ca^2+^ flux across the chloroplast envelope and participates in drought stress response [188]. Other important players include the calcium-sensing receptor (CAS; Figure 2; PDB: 7DD7, animal structure schematically used for visualization due to the lack of plant PDB data) and GLRs (Figure 2; PDB: 6385) [189,190,191]. Nevertheless, these channels and transporters are mainly involved in forming plastidial Ca^2+^ transients and regulating important chloroplast functions such as photosynthesis [43].

## 4. Similarities of Inter-Organellar Ca^2+^ Homeostasis in Plants and Animals

Inter-organellar Ca^2+^ homeostasis in plant and animal cells exhibits several similarities, despite their distinct evolutionary paths. Both systems rely on a complex network of Ca^2+^ channels, pumps, and exchangers to maintain precise spatiotemporal control over intracellular Ca^2+^ concentrations. The ER serves as an important intracellular Ca^2+^ store in both plant and animal cells [6,192], with Ca^2+^-ATPases actively pumping Ca^2+^ into the ER lumen. However, this aspect is not sufficiently understood in plant cells [183]. Mitochondria play a crucial role in modulating cytosolic Ca^2+^ signals and shaping Ca^2+^ microdomains in both systems [32,33]. Membrane contact sites between organelles, particularly between the ER and mitochondria and between ER and lysosomes, facilitate efficient Ca^2+^ transfer and fine-tuned regulation of cellular processes in both plant and animal cells [37,38,40,48,193]. Additionally, both systems utilize voltage-gated Ca^2+^ channels for rapid Ca^2+^ influx in response to membrane potential changes [27,39,71]. The coordinated efforts of these organelles and their associated proteins are critical for maintaining Ca^2+^ homeostasis, which is essential for cell survival, growth, and response to environmental stimuli in both systems [194,195]. As an example, both plant and animal TPCs function as intracellular cation channels that integrate voltage, Ca^2+^, and ligand signals to regulate Ca^2+^-dependent processes, including stress responses and organellar communication, with structural similarities such as dual Shaker-like domains enabling their conserved role in modulating cytosolic Ca^2+^ dynamics [71,171,196]. While plant TPCs (e.g., Arabidopsis AtTPC1) primarily mediate vacuolar Ca^2+^ waves during abiotic stress and immune signaling, animal TPCs (especially TPC1 and TPC2) similarly govern endolysosomal Ca^2+^ release for trafficking and metabolic regulation, highlighting their shared evolutionary origin as Ca^2+^-regulated gatekeepers of intracellular compartments [71,197,198].

## 5. Differences in Inter-Organellar Ca^2+^ Homeostasis in Plants and Animals

The regulation of Ca^2+^ homeostasis plays a pivotal role in both plant and animal cellular processes, but significant differences exist between these kingdoms. In plants, Ca^2+^ signaling has evolved to be more direct and specialized compared to animals [199,200]. While animals possess diverse Ca^2+^-influx mechanisms at the plasma membrane, plants have experienced a loss of diversity in this area [200]. For example, L-type and T-type voltage-gated Ca^2+^ channels are crucial for intracellular Ca^2+^ regulation in animal cells but are absent in plant cells, which have developed different Ca^2+^ signaling mechanisms, utilizing specialized channels like TPC, CNGCs, and GLRs to regulate intracellular Ca^2+^ homeostasis [199,200]. Plants have developed unique Ca^2+^-binding proteins, such as CDPKs and CDPK-related kinases (CRKs), which are absent in animals [200,201]. The evolution of the Ca^2+^-storing vacuole in plants provides additional possibilities for regulating cytosolic Ca^2+^ influx. Plants lack specialized muscle and nerve tissue-specific Ca^2+^ signaling genes found in animals, such as RyRs [200]. However, basic cellular Ca^2+^ homeostasis mechanisms such as MCUs and TPCs are conserved between plants and animals [27,32,38,188]. These differences in Ca^2+^ homeostasis reflect the distinct evolutionary pressures and environmental adaptations faced by plants and animals, particularly in response to abiotic stresses like temperature fluctuations [186]. As a direct example, animal TPCs are predominantly localized in endolysosomal membranes and are primarily activated by NAADP and PI(3,5)P_2_ to regulate Ca^2+^ signaling for vesicle trafficking and disease pathways, whereas plant TPCs such as AtTPC1 reside in vacuolar membranes and depend on voltage and cytosolic Ca^2+^ for stress-induced Ca^2+^ waves during immune responses [198,202]. Structural differences include EF-hand domains in plant TPCs enabling direct Ca^2+^ regulation, absent in animal TPCs, which instead exhibit NAADP-mediated activation and amplify signals via Ca^2+^-induced Ca^2+^ release from the ER [65,198].

## 6. Inter-Organellar Ca^2+^ Regulation in Animal and Plant Cells

Intracellular Ca^2+^ regulation is crucial for cellular signaling in both plants and animals, involving complex inter-organellar relationships. In animals, the ER–mitochondria interface plays a pivotal role in Ca^2+^ transfer, with optimal efficiency at a distance of about 20 nm [203]. Similarly, also in plants, Ca^2+^ fluxes between organelles like mitochondria, ER, Golgi bodies, and chloroplast are essential for Ca^2+^ signaling [204,205,206,207,208,209]. However, plants lack direct homologues of mammalian RyRs in the ER, suggesting different mechanisms. The plasma membrane–ER interface is critical in both systems for lipid and Ca^2+^ homeostasis [210,211,212,213]. In plants, the vacuole is a major Ca^2+^ store, unlike in animals where the ER is predominant [204]. In particular, TPCs should be highlighted, which are currently discussed as potential secondary regulators of intracellular Ca^2+^ via the interorganellar crosstalk between endo-lysosomes and ER [37,39,48] but also the vacuole and ER in plant cells [214]. In addition, the interaction between mitochondria and (endo)lysosomes plays an important role in intracellular Ca^2+^ homeostasis and mitochondrial Ca^2+^ dynamics [215,216]. Also, peroxisomes can play an important role in intracellular Ca^2+^ regulation by functioning as Ca^2+^-buffering organelles that maintain basal Ca^2+^ levels around 600 nM and can increase up to 2.4 µM upon stimulation in both animal and plant cells [217,218]. Only recently it was demonstrated that peroxisomes take up Ca^2+^ during cytosolic Ca^2+^ increases and exhibit beat-to-beat calcium uptake in cardiomyocytes, positioning them as crucial contributors to cellular Ca^2+^ homeostasis and excitation–contraction coupling [217]. The importance of the exact interplay of organelles in a cell was only recently elucidated in the organelle interactome study by Valm et al. [219]. There, a multispectral imaging method to study the interactions among six membrane-bound organelles in live cells was developed, revealing their spatial and temporal relationships and how these change under different conditions. This approach provides a powerful tool and new possibilities for understanding cellular organization and dynamics such as inter-organellar, intracellular Ca^2+^ regulation. Overall, while both plant and animal systems utilize Ca^2+^ as a secondary messenger, the specific inter-organellar interactions and regulatory mechanisms appear to distinctly differ between plants and animals. Table 1 compares well-characterized inter-organellar Ca^2+^ regulation, divided into kingdom, interacting organelles, specific functionality, and representative literature.

## 7. Excursus: Intracellular Ca^2+^ Regulation in Bacteria

While bacteria lack traditional membrane-bound organelles found in eukaryotes, they possess sophisticated Ca^2+^ transport systems that regulate intracellular Ca^2+^ homeostasis between different cellular compartments. Bacterial Ca^2+^ regulation involves specialized P-type ATPases such as LMCA1 from *Listeria monocytogenes*, which transports a single Ca^2+^ ion across the plasma membrane with unique mechanistic properties distinct from eukaryotic systems [220,221]. Recent studies have identified voltage-gated Ca^2+^ channels like CavMr in *Meiothermus ruber* that represent evolutionary links between bacterial and mammalian Ca^2+^ channels [222]. Bacteria also utilize Ca^2+^-binding proteins and Ca^2+^ leak channels, such as CalC in *Pseudomonas aeruginosa*, that mediate rapid Ca^2+^ transients in response to environmental stimuli and regulate gene expression controlling virulence factors and biofilm formation [223,224]. Additionally, some bacteria store Ca^2+^ in specialized compartments like acidocalcisomes, which contain Ca^2+^-PO_4_^3−^ complexes that function in Ca^2+^ homeostasis and potentially facilitate Ca^2+^ deposition for biofilm matrix mineralization [223,225]. These findings demonstrate that bacterial Ca^2+^ regulation represents a fundamental signaling mechanism that predates eukaryotic evolution and plays critical roles in bacterial survival, pathogenesis, and environmental adaptation. However, similar to plant cells, the extent of research regarding intracellular Ca^2+^ regulation lags behind that of animal cells.

## 8. Discussion and Conclusions

This review highlights the complex mechanisms of inter-organellar Ca^2+^ homeostasis in plant and animal systems, emphasizing the critical roles of various channels and transporters. Recent advancements in structural biology, imaging techniques, and advanced microscopic methods have significantly enhanced our understanding of these processes [19,21,22,23,226,227]. The complex interplay between organelles, particularly the ER, mitochondria, and endo-lysosomes, underscores the importance of membrane contact sites in facilitating efficient Ca^2+^ transfer [35,36,37,39]. The discovery of novel proteins like PDZD8 in mammalian neurons has shed light on the molecular basis of these interactions [40]. Additionally, the store-operated Ca^2+^ entry pathway, mediated by STIM and ORAI proteins, has emerged as a crucial mechanism for maintaining long-term Ca^2+^ homeostasis and signaling [41,42]. In plant systems, the presence of chloroplasts adds another layer of complexity to Ca^2+^ signaling networks, particularly in response to environmental stimuli [43]. Future research should focus on elucidating the precise spatiotemporal dynamics of Ca^2+^ signaling and its implications in various physiological and pathological conditions. This knowledge could pave the way for novel therapeutic interventions targeting Ca^2+^ homeostasis in both plant and animal systems. Intracellular Ca^2+^ regulation in plant cells and bacteria remains significantly more cryptic than in animal cells due to fundamental differences in cellular architecture and signaling complexity. Obviously, bacteria predominantly lack traditional membrane-bound organelles and plants have evolved different Ca^2+^ signaling systems compared to animals, with simplified influx mechanisms and yet more complex sensor networks that require two to three times more Ca^2+^-binding protein species to achieve the same signaling specificity [199]. This is based on the predominant sessile nature of plants, which necessitates more sophisticated internal regulatory mechanisms to respond to environmental changes [228]. The current understanding of plant Ca^2+^ channels remains remarkably limited, with many key channels still genetically unidentified and their mechanistic basis not fully understood [229]. Unlike animals, where Ca^2+^ oscillation systems are well characterized through IP_3_Rs and RyRs, plants lack these identical, homologous proteins [199], relying instead on less well-studied alternative mechanisms which still leave important scientific questions unanswered, decades after their initial investigation [230,231]. Overall, not only plant and animal cells have developed distinct strategies for intracellular and inter-organellar Ca^2+^ regulation, but even different cell types within a single organism have evolved diverse Ca^2+^ homeostasis mechanisms. Nevertheless, the fundamental principles of intracellular Ca^2+^ regulation are conserved, revealing surprising similarities from bacterial cells to plants and even human cells.

## Figures and Tables

**Table 1:** Inter-organellar Ca^2+^ regulation in animal and plant cells.

Kingdom	Organelles	Functionality	References
Animal	ER—mitochondria	Spatial Ca^2+^ transfer (IP_3_R)	[203]
Animal	Plasma membrane—ER	Lipid- and Ca^2+^ homeostasis (ORAI, IP_3_R)	[210,211]
Animal	Endolysosomes—ER	Spatial Ca^2+^ transfer (TPC)	[37,39,48]
Animal	Endolysosomes—mitochondria	Intracellular and mitochondrial Ca^2+^ dynamics (TRPML)	[215,216]
Animal	ER—Golgi	Interorganellar Ca^2+^ regulation (IP_3_R)	[207,208,209]
Plant	Chloroplast—mitochondria	Ca^2+^ signaling (MCU)	[204,205]
Plant	Plasma membrane—ER	Lipid- and Ca^2+^ homeostasis	[212,213]
Plant	Chloroplast—ER	Intracellular Ca^2+^ regulation	[206]
Plant	Vacuole—ER	Vacuolar Ca^2+^ buffering and spatial Ca^2+^ transfer (TPC)	[214,216]

## Data Availability

No new data were created or analyzed in this study.
